# Evaluation of a scoring system to assess proficiency in cerebral angiography for neuroendovascular surgery education

**DOI:** 10.1016/j.heliyon.2023.e13249

**Published:** 2023-01-27

**Authors:** Kouichi Misaki, Tomoya Kamide, Takehiro Uno, Taishi Tsutsui, Iku Nambu, Mitsutoshi Nakada

**Affiliations:** Department of Neurosurgery, Kanazawa University School of Medicine, Ishikawa, Japan

**Keywords:** Cerebral angiography, Education, Scoring, Preparation, Attention, Skill

## Abstract

**Objective:**

Cerebral angiography is indispensable for endovascular neurosurgeons. However, there is no established system to evaluate the competency of trainees. We established a scoring system and statistically analyzed its characteristics.

**Methods:**

Endovascular neurosurgeons scored the operators of 177 cerebral angiography based on ten evaluation items. Preoperative explanation, device selection, and device assembly were classified as “preparation,” communication with the patient, radiation protection and angiography system as “attention,” and catheter operation, blood loss, procedure completion, and sheath insertion as “skill”. The sum of the scores were compared using the Mann-Whitney test according to the status of the operator (trainee (TR), neurosurgeon (NS), or endovascular neurosurgeon (EVNS)).

**Results:**

The highest average for each item was 0.89 for communication, and the lowest was 0.68 for catheter operation. The mean ± standard deviation of the total score was 7.82 ± 2.02, and scores by status were 7.08 ± 2.12 for TR, 8.32 ± 1.35 for NS, and 9.33 ± 1.20 for EVNS with significant differences among each status (p < 0.05). The sum scores of the preparation, attention, and skill sections also showed significant differences between each status except between NS and EVNS in the preparation section and TR and NS in the skill section (p < 0.05).

**Conclusions:**

There were significant differences in the total score between statuses, suggesting that the scoring system may be an indicator of proficiency in cerebral angiography. It was suggested that dividing each item into preparation, attention, and skill sections may indicate the characteristics of proficiency.

## 1 Introduction

Due to an increase in the performance of endovascular treatment, the frequency of its education has increased. Moreover, cerebral angiography has been reported to be an essential procedure to learn [[1], [2], [3], [4]]. Nevertheless, education should be tailored to the proficiency of trainees, and vague procedural training avoided. However, there has been no report of a validated score for the proficiency of cerebral angiography as reported in general surgery [1,5]. Pathophysiology, device preparation, and patient communication skills are an important part of physician education in addition to procedural skills [4,6,7]. Attention to risky behavior and radiation exposure prevention is also an important factor, but no evaluation method covering all of them has been reported. A broader evaluation will increase the number of evaluation items, but past reports have generally evaluated about 10 items [1,5].

In this study, we established and reviewed ten items to evaluate the proficiency of operators in the performance of angiography to consider angiography content and devices according to the patient's pathological condition (preparation), perform angiography while paying attention to patient communication, low radiation exposure, and angiography system (attention), and perform scheduled procedures without complications (skill). We statistically analyzed these scores among three statuses: residents and fellows (trainees; TR), physicians with only neurosurgery certification (neurosurgeons; NS), and physicians with both neurosurgery and endovascular treatment certification (endovascular neurosurgeons; EVNS), and analyzed whether this evaluation system is appropriate as an indicator of the proficiency and characteristics of the operators.

## 2 Methods

This is a retrospective analysis of operator scores for diagnostic cerebral angiography by directly supervising EVNS in person. Inclusion criteria were diagnostic cerebral angiography without endovascular treatment performed at Kanazawa University from January 2020 to April 2021. Exclusion criteria were 1 angiography with balloon occlusion test, 2 spinal angiography, and 3 angiography without scoring assessment due to absence of EVNS. Total of 177 diagnostic cerebral angiographies that scored by EVNS were reviewed. Cerebral angiography was performed by puncturing the femoral or brachial artery with a 4Fr sheath under local anesthesia without sedation. A 4Fr catheter (JB2 type for the femoral approach and Simmons type for the brachial approach) was advanced to the target blood vessels using a 0.035-inch guidewire. If the operator performed dangerous or repeated unsuccessful procedures, the EVNS was replaced.

There are ten evaluation items divided into three sections; “preparation” for preoperative explanation, device selection, and device assembly; “attention” for communication with the patient, radiation protection and angiography system; and “skill” for catheter operation, blood loss, procedure completion, and sheath insertion (Table 1). The preoperative explanation was evaluated to determine whether the operator could explain the puncture site and blood vessel to be imaged to the assistant or co-medical staff in times of time-out. The second item, device selection, assessed whether the operator could prepare sheaths, catheters, and guidewires according to the required examination. The third item, device assembly, assessed whether the operator could prepare pressure lines and contrast injectors without air inclusion. In the fourth item, communication with the patient, we assessed whether the operator could relieve anxiety by talking to the patient, especially before local anesthesia and contrast injection. In the fifth item, radiation protection, we assessed whether the radiation shield and flat panel were properly installed and whether radiation exposure protection was appropriately performed. The sixth item, angiography system, was evaluated to determine whether the operator could operate the imaging system appropriately without hesitation. The seventh item, catheter operation, was evaluated to determine whether the catheter could reach the target vessel appropriately, and in cases with difficulty due to vessel tortuosity, three repeated failures were scored as zero. The eighth item, blood loss, was assessed to determine whether the operator could minimize the amount of blood on gloves and sheets. The ninth item, procedure completion, assessed whether the operator did or did not perform a dangerous procedure or needed to be replaced by a senior doctor if it was determined that the operator could not continue the angiography. The tenth item, sheath insertion, reviewed whether the operator could insert the sheath with no more than two punctures and in the correct position with no dissection. One item was assigned 1 point and zero points were given if there was a problem with an item. If all 10 items were passed, the score was calculated out of 10 points. Operators were informed in advance that they would be assessed using the system described above.

**Table 1:** Scoring items for cerebral angiography.

Classification	Item	Detail
Preparation	Preoperative explanation	Explanation of puncture site and blood vessel to be imaged during time-out
	Device selection	Exact device selection of sheath, catheter and guidewire
	Device assembly	Device assembly of contrast injector and pressure line
Attention	Communication with patient	Sufficient communication with patient to relieve anxiety
	Radiation protection	Proper placement of radiation shield and flat panel
	Angiography system	Proper and smooth use of angiography system
Skill	Catheter operation	Exact operation of catheter within three same failures
	Blood loss	Minimal blood loss to the gloves and sheets
	Procedure completion	Completion of entire procedure without replacement by senior physician
	Sheath insertion	Smooth sheath insertion within two punctures

All statistical analyses were performed using IBM SPSS Statistics, version 19.0 (SPSS, Inc., Chicago, IL, USA). The correlation between each score was examined using Spearman's rank correlation test to analyze factors with a strong correlation. The sum scores were also compared using the Mann-Whitney test separately by operator's status (TR, NS, and EVNS). We did not use Pearson's correlation, *t*-test, and ANOVA due to non-normally distributed factors.

This study was reviewed and approved by the institutional ethics committee of Kanazawa University (No. 2021-095). Informed consent was obtained from all participants.

## 3 Results

The operators performing cerebral angiography were six TR (two to seven years of experience as physician with 0 to 79 (mean 44) prior experiences of angiography) in 100 cases, three NS (ten to 12 years of experience with 147 to 254 (mean 197) prior experiences of angiography) in 41 cases, and three EVNS (13–21 years of experience with 251 to 1511 (mean 717) prior experiences of angiography). Angiography revealed 92, 35, 19, 12, 11, and eight cases of cerebral aneurysm, arteriosclerotic disease, brain tumor, dural arteriovenous fistula, moyamoya disease, and arteriovenous malformation, respectively (Table 2). Of the 177 angiographies performed, one (0.6%) suffered from a puncture hematoma, but no abnormal neurological symptoms occurred.

**Table 2:** Demographic data of cerebral angiography.

	All	Status		
		TR	NS	EVNS
Age	64 ± 15	64 ± 15	63 ± 13	66 ± 16
Female: Male	102 : 75	56 : 44	22 : 19	24 : 12
Disease				
Aneurysm	92	46	23	23
Atherosclerosis	35	26	8	1
Brain tumor	19	11	5	3
Moyamoya disease	12	2	2	8
Dural arteriovenous fistula	11	8	3	0
Arteriovenous malformation	8	7	0	1
Sum	177	100	41	36

EVNS endovascular neurosurgeon, NS neurosurgeon, TR trainee, Values are n (%) or mean ± standard deviation.

**Fig. 1:**
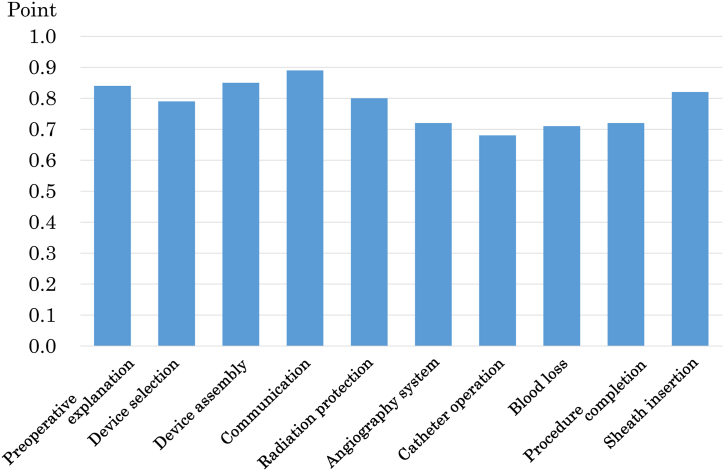
The bar graph shows the average scores for the evaluated items. The average score ranged from 0.68 to 0.89, and the lowest and highest were catheter operation and communication, respectively.

**Fig. 2:**
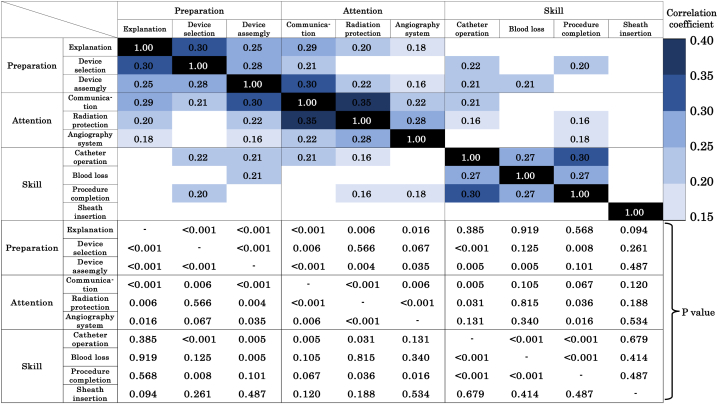
The correlation coefficients only for those with significant differences in the ten evaluation items. Significant correlations were found among each item of the preparation, attention, and skill sections with correlation coefficients of 0.25–0.30, 0.22–0.35, and 0.27–0.30, respectively. No item correlated with sheath insertion.

The highest average for each item was 0.89 for communication with patient, and the lowest was 0.68 for catheter operation (Fig. 1). Fig. 2 shows the correlation coefficients only for those with significant differences for each item and all p-values. Significant correlations were found among each item of the preparation, attention, and skill sections, with correlation coefficients of 0.25–0.30, 0.22–0.35, and 0.27–0.30, respectively. However, no items correlated with sheath insertion, which is an evaluation item for the skill section.

**Fig. 3:**
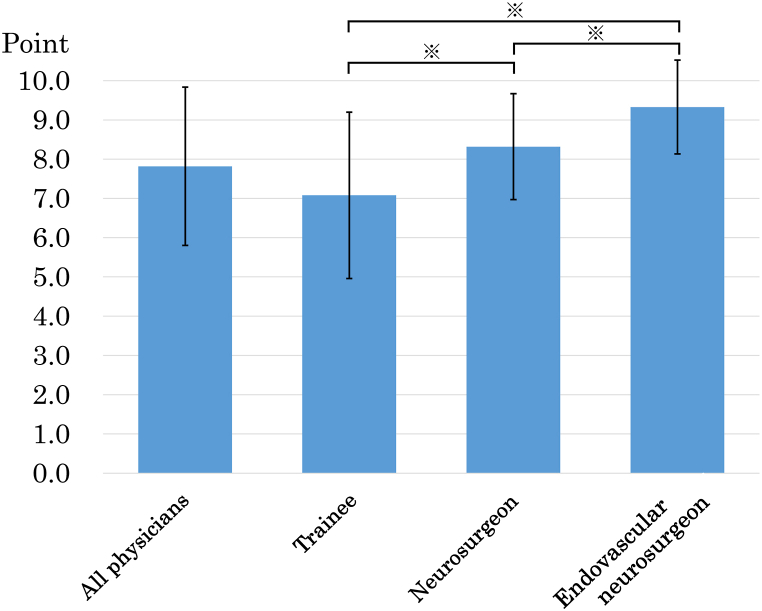
Bar graph showing the average total scores of the ten evaluated items. The average total scores were 7.82 ± 2.02 (mean ± standard deviation), 7.08 ± 2.12, 8.32 ± 1.35, and 9.33 ± 1.20, for all physicians, trainees, neurosurgeons, and endovascular neurosurgeons, respectively. ∗ indicates significant differences in the Mann-Whitney test.

**Fig. 4:**
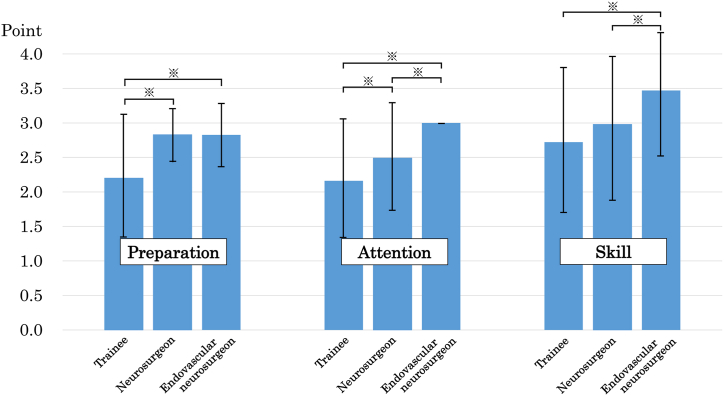
Bar graph showing the average sum scores of the trainees, neurosurgeons, and endovascular neurosurgeons in each of the three evaluation sections, including preparation, attention, and skill. ∗ indicates significant differences in the Mann-Whitney test.

The mean ± standard deviation of the total average score of all physicians was 7.82 ± 2.02, and the scores by status were 7.08 ± 2.12 for TR, 8.32 ± 1.35 for NS, and 9.33 ± 1.20 for EVNS, respectively. The Mann-Whitney test of the total scores showed significant differences between the groups (p < 0.05, Fig. 3, Table 3). The sum scores of the preparation, attention, and skill sections showed significant differences between all groups except between NS and EVNS in the preparation section and between TR and NS in the skill section (Fig. 4, Table 3).

**Table 3:** The evaluation system scores and the Mann-Whitney test p-values.

	All	Status			p-value		
		TR	NS	EVNS	TR vs NS	NS vs EVNS	TR vs EVNS
Total points	7.82 ± 2.02	7.08 ± 2.12	8.32 ± 1.35	9.33 ± 1.20	0.002	<0.001	<0.001
Preparation	2.47 ± 0.82	2.20 ± 0.94	2.83 ± 0.38	2.83 ± 0.45	<0.001	0.745	<0.001
Attention	2.41 ± 0.83	2.16 ± 0.88	2.49 ± 0.78	3.00 ± 0.00	0.029	<0.001	<0.001
Skill	2.93 ± 1.06	2.72 ± 1.06	2.98 ± 1.01	3.47 ± 0.94	0.172	0.009	<0.001

EVNS endovascular neurosurgeon, NS neurosurgeon, TR trainee, Values are n (%) or mean ± standard deviation.

## 4 Discussion

This is the first report of a scoring system that can be used as an index of proficiency in cerebral angiography. We found significant differences in the average total score between the three groups of physicians (TR, NS, and EVNS). In Japan, the neurosurgery board exam requires two years of initial resident training followed by a minimum of four years of neurosurgical training at a designated specialty training facility. The exam consists of a written and oral examination with a pass rate of approximately 70% [8]. The exam for the certification of neuroendovascular treatment is a rigorous examination that requires a specialist in neurosurgery, neurology, radiology, or internal medicine to have experience in 100 cases of endovascular treatment and 200 cases of cerebral angiography with a pass rate of approximately 50% [8]. Although there is no numerical index for proficiency of cerebral angiography, we consider that this evaluation system indicates proficiency level of angiography by following reasons; 1 high knowledge and skills are required to pass above tests, 2 a certain level of experience is necessary to maintain above certifications, 3 the number of angiography experiences increases as the certification increases in this study.

The present scoring system evaluated various characteristics, including preparation, attention, and technical skills. Previous reports on cerebral angiography education have also reported that sufficient cognitive training, including education on neurovascular anatomy, pathophysiology, and knowledge concerning devices, is important in addition to technical training [4]. Significant correlations were found between the three items of preparation, including preoperative explanation, device selection, and device assembly. These items can be fully prepared for a long time before angiography is initiated. There were significant correlations between the three preparation items, suggesting that physicians tended to prepare all three items prior to angiography. There was a significant difference between TR and NS in the sum score of preparation, but there was no significant difference between NS and EVNS. This result indicates that the content of the preparation section should be learned before becoming an NS. Physicians with a low score in the preparation section lack cognitive training or a basic attitude toward angiography, and education centered on lectures rather than practical training is considered effective [4].

The three items of the attention section, including communication with the patient, radiation protection, and the angiography system, are all skills that are required to respond to various situations during angiography that cannot be completely mitigated at the preparation stage. Significant correlations were also found among the three items in the attention section. The communication item had the highest average score among the ten items, and this score is considered to easily increase because communication with patients does not require specialized knowledge about radiation protection and the angiography system. However, communication ability was reported to correlate with patient satisfaction and treatment results and is therefore essential for physician education [6]. Radiation protection is an essential educational item in procedures that result in radiation exposure, and it tends to be neglected if the physician concentrates too much on the catheter or guidewire displayed on the monitor [9]. Since the item of radiation protection highlights the ability to pay attention to the surrounding conditions other than the monitor, the radiation protection score strongly correlates with the communication score. In our hospital, the angiography system is mainly operated by physicians rather than radiological technicians, and the operator moves the table, changes the irradiation range and imaging conditions and sets a roadmap to obtain the best image for endovascular treatment. The angiography system score was the lowest among the attention sections because it requires a high ability to judge according to the angiography situation, in addition to the expertise on the switch of the angiography system. The fact that the sum scores of the attention section had significant differences between both TR and NS, and NS and EVNS indicates that it takes a long time to acquire the ability to respond to various situations during angiography.

The four skills section items, including catheter operation, blood loss, completion of the procedure, and sheath insertion, review whether the procedure was performed without any issues. The lowest average score among the ten items was for catheter operation, which is a unique technique that cannot be obtained through the performance of open surgery. It has already been reported that physicians perform better surgery when there is smaller blood loss, and it is considered that the same applies to angiography and endovascular treatment [10]. The item procedural completion assesses whether the physician needed to be replaced by a senior physician because of dangerous or unsuccessful repeated procedures. It is reported that inexperienced physicians are more likely to perform dangerous and repeated procedures [4,11]. Since the average score of this item is 0.72, indicating EVNS were replaced in about 30% of the angiographies, the overall complication rate in the current study is lower than that in previous reports [12]. Angiography and endovascular treatment need to avoid complications specific to catheter operation that differ from open surgical procedure [[13], [14], [15]]. In cases of difficulty in angiography due to vascular tortuosity or non-cooperation of patients, the NS were replaced by EVNS, reducing the ‘skill’ score. This was considered the reason for the lack of a significant difference in the sum scores of skills between TR and NS. Accurate assessment of maturity requires an objective indicator of procedural difficulty. Only the sheath insertion item did not correlate with the other items. This was likely because the puncture was sufficiently mastered by training in the operating room and ward previously.

The usefulness of this scoring system is considered to be: 1 to score physician inexperience (preparedness, attention, skill, or overall) in the curriculum, 2 to score physician growth over time, 3 setting cut-off points and goals to pass the NS and EVNS exams, 4 to identify score characteristics of physicians with adverse events for proactive prevention.

Simulations have been reported to be useful for the education of trainees [1,3,[16], [17], [18]]. However, the greatest limitations of simulator-based education are the tactile discrepancy and high cost of the simulator [1,19]. Because angiography is performed with local anesthesia, responsiveness such as communication with patients cannot be improved by simulation [6]. However, since animal models and simulations have the advantage of repeatability, combining simulations with scoring systems may lead to more effective education [20,21]. The disadvantage of clinical education over simulator-based education is the case selection bias shown in Table 2 with heterogeneous case distribution among the three certifications.

Limitations of this study are as follows; the contents of this report are a retrospective analysis, and the number of cases and evaluated physicians was small. The difficulty of the different procedures cannot be scored, and the objectivity of the evaluation may not be uniform. The evaluation score was judged as zero or one, not in five steps such as when using the OSATS or Likert scale [5,17]. There is no comparison or control group before and after this scoring system, and no verification of EVNS opinion of other institutions or concordance of scores between EVNS, so further validation is needed. Many of the assessments in this study are practical and may not cover a wider range of cognitive knowledge. Because we notified participants about the scoring system prior to the study commencing, the negative effects of mental atrophy and the inability of trainees to demonstrate their original abilities should be considered.

## 5 Conclusions

The scoring system may be an indicator of proficiency in cerebral angiography because there were significant differences in the total score between the three statuses. It was suggested that dividing each item into preparation, attention, and skill sections may indicate the characteristics of proficiency.

## Production notes

### Author contribution statement

Kouichi Misaki: Conceived and designed the experiments; Performed the experiments; Analyzed and interpreted the data; Wrote the paper.

Tomoya Kamide: Performed the experiments; Contributed reagents, materials, analysis tools or data.

Takehiro Uno: Analyzed and interpreted the data.

Taishi Tsutsui: Performed the experiments; Wrote the paper.

Iku Nambu: Contributed reagents, materials, analysis tools or data.

Mitsutoshi Nakada: Conceived and designed the experiments.

### Funding statement

Dr. Kouichi Misaki was supported by Japan Society for the Promotion of Science [21K09120].

### Data availability statement

Data included in article/supp. material/referenced in article

### Declaration of interest’s statement

The authors declare no competing interests.
